# Rutaecarpine suppresses the proliferation and metastasis of colon cancer cells by regulating the STAT3 signaling

**DOI:** 10.7150/jca.66177

**Published:** 2022-01-01

**Authors:** Shixin Chan, Rui Sun, Xucan Tu, Manyu Guo, Qianqian Yuan, Zhen Yu, Zhenglin Wang, Shaocheng Hong, Wei Han, Bingbing Zou, Zeng Li, Huabing Zhang, Wei Chen

**Affiliations:** 1 Department of General Surgery, The First Affiliated Hospital of Anhui Medical University, Hefei, 230022, China; 2 Department of Biochemistry & Molecular Biology, Metabolic Disease Research Center, School of Basic Medicine, Anhui Medical University, Hefei, 230032, China; 3 Anhui Provincial Institute of Translational Medicine, Hefei, 230022, China; 4 Department of General Medicine, The First Affiliated Hospital of Anhui Medical University, Hefei, 230022, China; 5 The First Clinical Medical College of Anhui Medical University, Hefei 230032, China; 6 Department of Gastroenterology, The First Affiliated Hospital of Anhui Medical University, Hefei, 230022, China; 7 Department of Gastrointestinal Surgery, General Surgery, The First Affiliated Hospital of Anhui Medical University, Hefei, 230022, China; 8 School of Pharmacy, Anhui Medical University, Hefei, 230032, China

**Keywords:** colorectal cancer, Rutaecarpine, STAT3, NF-κB, proliferation, apoptosis

## Abstract

Colorectal cancer (CRC) is a malignant disease that is a serious threat to human health. Rutaecarpine (RUT) is an important bioactive alkaloid of Evodia rutaecarpa. According to previous studies, RUT suppressed the proliferation of several human tumors. However, its role in colorectal tumorigenesis remained unknown. The aim of the present study was to determine the functions of RUT in CRC. Here, we have demonstrated that RUT inhibited the proliferation, migration and invasion of CRC cells* in vitro*. Further, RUT was found to induce the apoptosis of CRC cells. Mechanistically, RUT decreased the phosphorylation levels of NF-κB and STAT3. Moreover, treatment with RUT upregulated the expression of cleaved-Caspase3 and downregulated the expression of Bcl-2 in CRC. In addition, our findings suggested that RUT inhibited the growth and lung metastasis of CRC Cells *in vivo*. Based on immunofluorescence analysis, the expression of Ki67 was downregulated while that of cleaved-Caspase3 was upregulated in RUT-treated tumors compared with control-treated tumors. Taken together, our findings indicate that RUT can inhibit the proliferation and migration of CRC cells, and induce the apoptosis of CRC cells by inactivating NF-κB/STAT3 signaling. Our study highlights the potential clinical application of RUT for the treatment of CRC.

## Introduction

Colorectal cancer (CRC) is the third most common malignant tumor, has the second highest fatality rate worldwide, and has long threatened human life and health 1. The number of patients with CRC is projected to expand to 2.2 million globally by 2030, and the number of deaths will be more than 1.1 million 2. CRC is associated with multiple causes, including genetic factors, environmental factors, and dietary structure 3. However, the occurrence and development of CRC is a multi-stage and multi-step biological behavior process involving multiple factors and the activation of multiple signaling pathways, such as Wnt, NF-κB, STAT3, PI3K and Notch signaling pathway 4-8. With the improvement and enrichment of methods, such as surgery, chemotherapy, radiation therapy, and immunotherapy, the 5-year survival rate of early-stage CRC can reach 90% 9. However, the drug resistance exhibited by some CRC patients to conventional chemotherapy makes the treatment effect unsatisfactory 10. Therefore, discovering novel therapeutic drugs for the clinical treatment of CRC is urgently needed.

**Figure 1:**
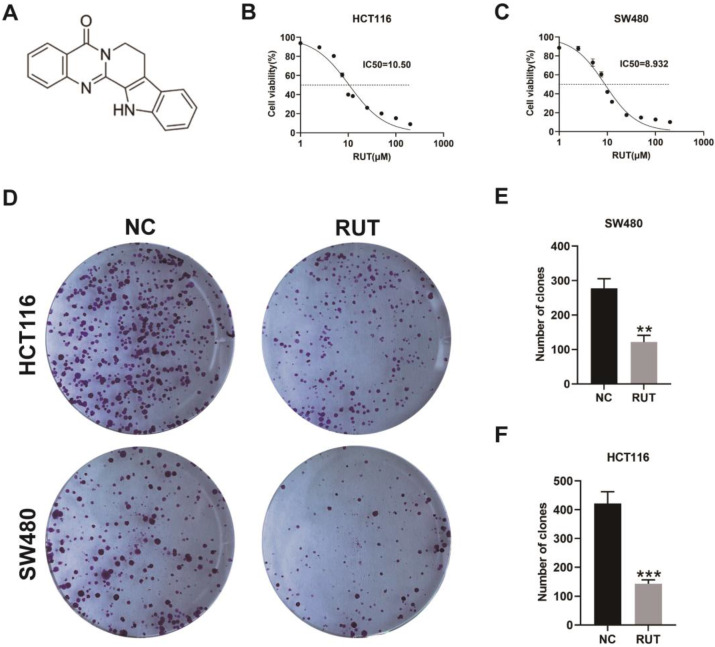
** RUT decreased the viability of CRC cells and inhibited the proliferation of CRC cells.** (A) The chemical structure of rutaecarpine. (B-C) Cell viability assays showed that RUT (1 μM, 2.5 μM, 5 μM, 7.5 μM, 10 μM, 12.5 μM, 25 μM, 50 μM, 100 μM, 200 μM for 24 h, respectively) suppressed the viability of CRC cells. The IC50 values of RUT in both cells (HCT116 and SW480) are indicated. (n=3). (D) The effect of RUT on colony formation. (n=3). (E-F) The bar graphs indicate mean colony numbers ± standard deviation (SD) formed by HCT116 and SW480 cells treated with or without 5 μM RUT for 10 days. **P < 0.01, ***P < 0.001.

In recent years, growing evidence has suggested that many extracts from natural plants are effective against cancers. The alkaloid component, rutaecarpine (RUT, Figure 1. A), is the main extract from the traditional Chinese medicine, Evodia rutaecarpa, which has been proven to have many biological characteristics 11, and plays critical roles in cardiovascular, nervous system, inflammatory and tumor diseases. According to a previous study, RUT can protect against hypertensive myocardium by inhibiting the NOX4 pathway 12. RUT can also inhibit endoplasmic reticulum stress mediated by the Caspase-12 and NF-κB pathways, and improve sepsis-induced peritoneal macrophage apoptosis and inflammatory response 13. Moreover, RUT can act on a variety of tumor cells, including lung cancer 14, prostate cancer 15, 16, thyroid cancer 17, breast cancer 18, gastric cancer 19, and liver cancer 20, 21 through different mechanisms of action, including: 1) changing the cell cycle and inhibiting proliferation; 2) inducing cell apoptosis; 3) reducing angiogenesis; and 4) affecting invasion and metastasis 22. However, only few reports have been published on the therapeutic effect of RUT on CRC 23.

In the present study, we demonstrated that RUT inhibited the proliferation and invasion of CRC cells and induced the apoptosis of CRC cells *in vitro* and *in vivo* by regulating the NF-κB/STAT3 pathway. Such findings provide a new insight into the potential use of RUT for the treatment of CRC.

## Materials and methods

### Reagents and antibodies

RUT (purity, 98%) was purchased from Aladdin, China. 3-(4,5-dimethylthiazol-2-yl)-2,5-diphenyltetrazolium bromide (MTT) was obtained from Solarbio, China. Annexin V-FITC/PI apoptosis kit was purchased from Bestbio, China. Dimethyl sulfoxide (DMSO) was provided by Sigma-Aldrich. Antibodies against NF-κB, p-NF-κB, STAT3, and p-STAT3 were purchased from Cell Signaling Technology. Other antibodies used in this study were: Bcl-2 (Affinnity biosciences), cleaved-Caspase3 (Abclonal), CDK4 (Proteintech) and GAPDH (Santa). The secondary antibody used in this study was HRP conjugated goat anti-mouse/rabbit (Abclonal). The electrochemiluminescence (ECL) Western blotting detection reagents were obtained from Beyotime.

### Cell culture

The CRC cell lines, HCT116 and SW480, were obtained from American Typical Culture Center. Cells were incubated at 37 °C with 5% CO_2_ in Dulbecco's modified Eagle's medium (DMEM, Gibco) supplemented with fetal bovine serum (FBS, Lonsera, Australia), penicillin (100 U/ml) and streptomycin (100 mg/ml, Biosharp, China). All cells were grown at 37 °C in a humidified atmosphere with 5% CO_2_.

### Cell viability assay

The CRC cells were seeded in 96-well plates at a density of 2 × 10^3^ per well, and cultured in incubators. After the cells to adhered to the wall of the plates, the experimental group was treated with a pre-designed drug concentration gradient while the solvent group was treated with the same amount of DMSO. After incubation at 37 ℃ for 24 h, 25 μl MTT solution was added to each well, and returned to the incubator for a 1 h incubation. Thereafter, the medium was discarded and 100 μl DMSO was added to each well. The plates were incubated for 10 min. Finally, the absorbance value of each well at OD490 nm was measured using a microplate analyzer.

### Colony formation assay

The cells were inoculated in 6-well plates at 1000 cells per well. After the cells were attached, the experimental and control group were treated with 5 μM RUT and the same volume of DMSO, respectively. Complete DMEM containing 10% FBS and 1% double antibody (streptomycin and penicillin) was replaced every three days. RUT and DMSO were added to the wells to ensure the maintenance of drug concentration. After 10 days, the adherent cells were fixed with methanol for 10 min. Finally, the cells were visualized by staining with 0.1% crystal violet at room temperature, and then washed twice with PBS.

### Wound healing assay

The CRC cells were evenly inoculated into 6-well plates at a density of 5 × 10^5^ cells per well. After the cells adhered to the wall of the plates, the experimental group and the control group were treated with RUT (5 μM) and equal volume of DMSO, respectively, and cultured in incubators. The scratches were initiated when the monolayer cells approached fusion. The migration of cells was recorded using an inverted microscope at 0 h, 24 h, and 48 h, respectively. The distance of cell migration was determined using ImageJ software.

### Transwell assay

A total of 5 × 10^4^ CRC cells treated or not treated with RUT were suspended in 100 μl serum-free medium and added to the transwell upper compartment, respectively. The lower compartment was filled with 500 μl medium containing 20% FBS. After incubation at 37 ℃ for 24 h, the cells at the bottom of the membrane were fixed with 4% formaldehyde solution at room temperature for 20 min, stained with 0.1% crystal violet at room temperature for 30 min, and washed twice with PBS. Cell counts in five different areas were recorded using an inverted microscope.

### Apoptosis assay *in vitro*

To determine the effect of RUT on cell apoptosis, we carried out flow apoptosis experiments. The CRC cells were evenly seeded into 6-well plates at a density of 5 × 10^5^ per well. After the cells were attached to the wall, RUT (5 μM) and DMSO were added to the experimental group and the control group, respectively, and incubated in an incubator for 24 h. The medium was then discarded, and the cells were washed 3 times with warm PBS. Thereafter, the cells were digested with trypsin without EDTA, washed twice with cold PBS, and then resuspended with the Annexin V binding solution. The Annexin V-FITC dye was added, and the cells were incubated at 4 ℃ in the dark for 15 min. Finally, propidium iodide (PI) dye was added and incubated at 4 ℃ in the dark for 5 min. Cell apoptosis was detected using a flow cytometer.

### Western blotting

For western blot analysis, total protein was extracted from the cells using RIPA (Beyotime) buffer. The same amount of protein was separated via sodium dodecyl sulfate-polyacrylamide gel and transferred to polyvinylidene fluoride (PVDF) membrane. The PVDF membrane was sealed with 5% skimmed milk for 1 h at room temperature, and incubated overnight at 4 ℃ with primary antibodies NF-κB (1:1000), p-NF-κB (1:1000), STAT3 (1:2000), p-STAT3, (1:1000), Bcl-2 (1:1000), cleaved-Caspase3 (1:1000), CDK4 (1:2000) and GAPDH (1:1000), and then followed by HRP conjugated secondary antibody for 1 h at room temperature. The protein bands were quantitatively analyzed using ECL on a chemiluminescence apparatus.

### Nude mouse tumor formation assay and lung metastasis models

Five-week-old BALB/c male nude mice were purchased from Model Animal Research Centre of Nanjing University and raised in the SPF animal room. All animal testing procedures have been approved by the Animal Ethical Committee of Anhui Medical University and were carried out in strict accordance with the guidelines of the Animal Center of Anhui Medical University. A total of 2 × 10^6^ HCT116 cells were subcutaneously injected into the right flank of nude mice and tumorigenesis was observed. The tumor volume was 1/2 × length × width × width. When the tumor volume approached 100 mm^3^, 50 mg/kg RUT (dissolved in the solution containing 10% DMSO, 10% Tween80 and 80% NaCl) was injected into the tumor of experimental mice every two days, and the same volume of vehicle was injected into control mice. All mice were killed after 4 weeks and the tumors were collected for further analysis. A total of 5 × 10^6^ HCT116 cells were injected via the tail vein; thereafter, 60 mg/kg RUT (dissolved in solution containing 10% DMSO, 10% Tween80 and 80% NaCl) was injected intraperitoneally into experimental mice every two days, while the same volume of vehicle was injected into control mice. All mice were killed after 8 weeks and the tumors were collected for further analysis.

### Immunofluorescence staining

Colonic tumor tissues from nude mice were fixed in 10% formalin and embedded in paraffin, cut into 5 μm sections, and placed on adhesive slides. The slides were treated with xylene, ethanol, and then distilled water. Antigen repair was performed using EDTA. The sections were sealed with 5% normal goat serum at 37 ℃ for 30 min. The slides were incubated with the primary antibodies for Ki67 (Abclonal, 1:50) and cleaved-Caspase3 (Abclonal, 1:50) at 4 ℃ overnight, and then with the secondary antibody at 37 ℃ in the dark for 1 h. The sections were incubated with DAPI at room temperature in the dark for 10 min and observed under a microscope.

### Histopathological analysis

For histopathological analysis, lung tissues were fixed in 10% formalin and embedded in paraffin. After serially cutting, the tissue sections (5 μm) were stained with hematoxylin and eosin (HE) to confirm the presence of lung metastatic foci. Finally, the pathological changes were observed using a microscope.

### Statistical analysis

All experiments were repeated at least 3 times, and the data are expressed as mean ± SD. All data were analyzed using GraphPad Prism 9, and the differences between the two groups of data were compared using Student's *t* test. P < 0.05 was considered to indicate statistical significance.

## Results

### RUT inhibits the proliferation of CRC cells *in vitro*

Previous studies have demonstrated that RUT can inhibit the growth of a variety of tumors 16, 20. To determine the effect of RUT on the proliferation ability of CRC cells, we conducted the MTT assay and the colony formation assay. Based on the results, RUT significantly reduced the viability of CRC cells (HCT116 and SW480) in a concentration-dependent manner (Figure 1. B and C). In addition, RUT was demonstrated to have a better inhibitory effect than 5-fluorouracil at the same concentration (Fig. S1). According to the fitting formula, the IC50 values of RUT for HCT116 and SW480 were 10.50 μM and 8.93 μM at 24 h of RUT treatment, respectively. As a result, we selected the drug concentration of 5 μM to conduct the colony formation experiment. CRC cells treated with RUT had decreased colony-forming abilities compared with the control-treated cells (Figure 1. D-F). These results confirm that RUT can inhibit the proliferation of CRC cells *in vitro*.

### RUT suppresses the migration and invasion of CRC cells* in vitro*

**Figure 2:**
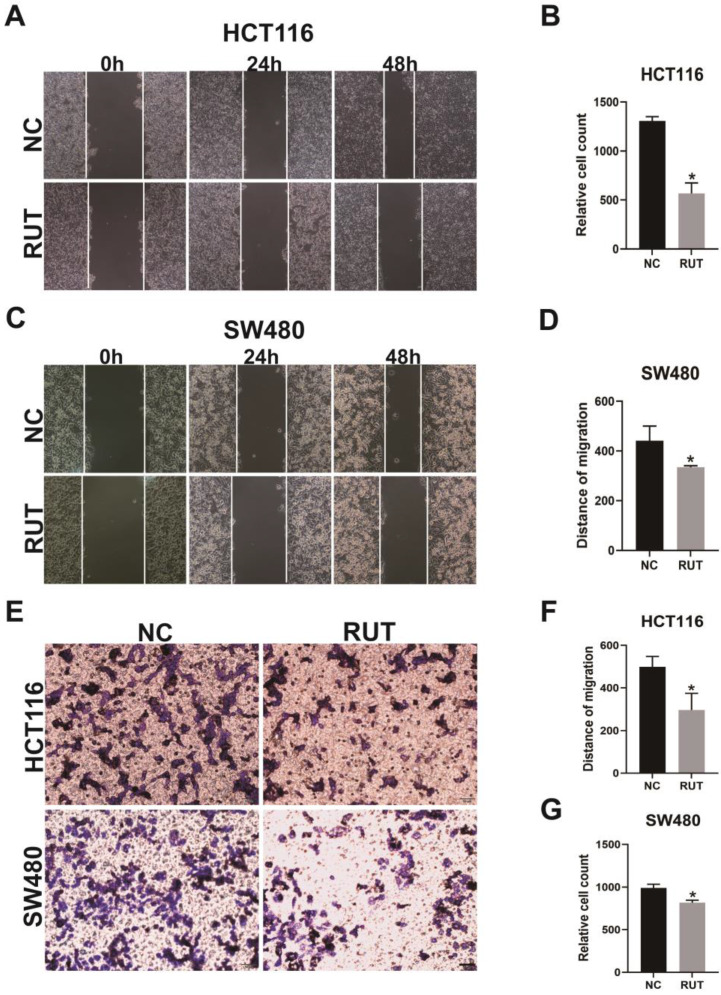
** Effects of RUT on the migration and invasion of CRC cells in the wound healing assay and transwell assay.** (A-B) The wound healing assays revealed that the migration ability of HCT116 cells treated with RUT (5 μM) was significantly decreased compared to that of control-treated cells. (n=3). (C-D) The wound healing assays revealed the migration ability of SW480 cells. (n=3). (E-G) The transwell assays revealed that RUT impaired the invasion ability of CRC compared with the control (Scale bar: 50 μm). *P < 0.05.

To further elucidate the roles of RUT in the migration and invasion of CRC cells, we performed wound healing assay and transwell assay. The wound healing assays revealed that RUT treatment decreased the migratory distance of CRC cells compared with that of cells treated with the control (Figure 2. A-D). Furthermore, our data confirmed that RUT significantly impaired the invasion ability of CRC cells compared with the control (Figure 2. E-G). These findings suggest that RUT suppresses the migration and invasion of CRC cells *in vitro*.

### RUT decreases the phosphorylation levels of NF-κB and STAT3 in CRC cells

**Figure 3:**
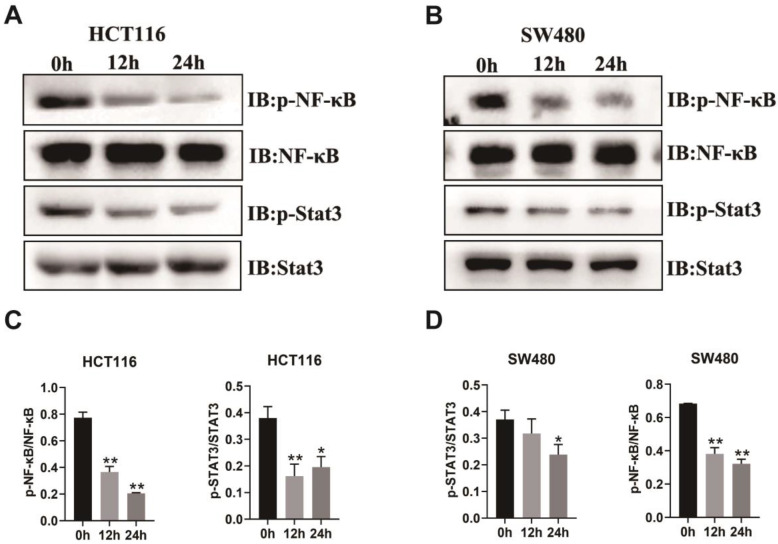
** Impact of RUT on the signaling pathway of CRC cells.** (A-B) RUT (5 μM) significantly decreased the level of phosphorylated NF-κB and STAT3 in HCT116 (A) and SW480 (B) cells after treatment for 24 h. (n=3). (C-D) Statistical results of the protein levels indicated in A and B. *P < 0.05, **P < 0.01.

The above results demonstrated that RUT inhibited the proliferation, migration, and invasion of CRC cells *in vitro*. To determine whether the inhibitory effect of RUT affects the protein levels of key pathway components of CRC, we carried out western blotting to analyze the alterations in the proliferation abilities of CRC cells. The transcription factors, NF-κB and STAT3, are particularly important in the development of CRC 5, 24-26. As revealed by western blotting, RUT treatment decreased the phosphorylation levels of NF-κB and STAT3 in both HCT116 (Figure 3. A) and SW480 cells (Figure 3. B). However, we did not observe changes in the phosphorylation levels of GSK-3β and P38 (Fig. S2). Taken together, these results demonstrate that RUT may suppress colorectal tumorigenesis by inhibiting the activation of NF-κB and STAT3.

### RUT induces CRC cell apoptosis and cleaved-Caspase3 expression

**Figure 4:**
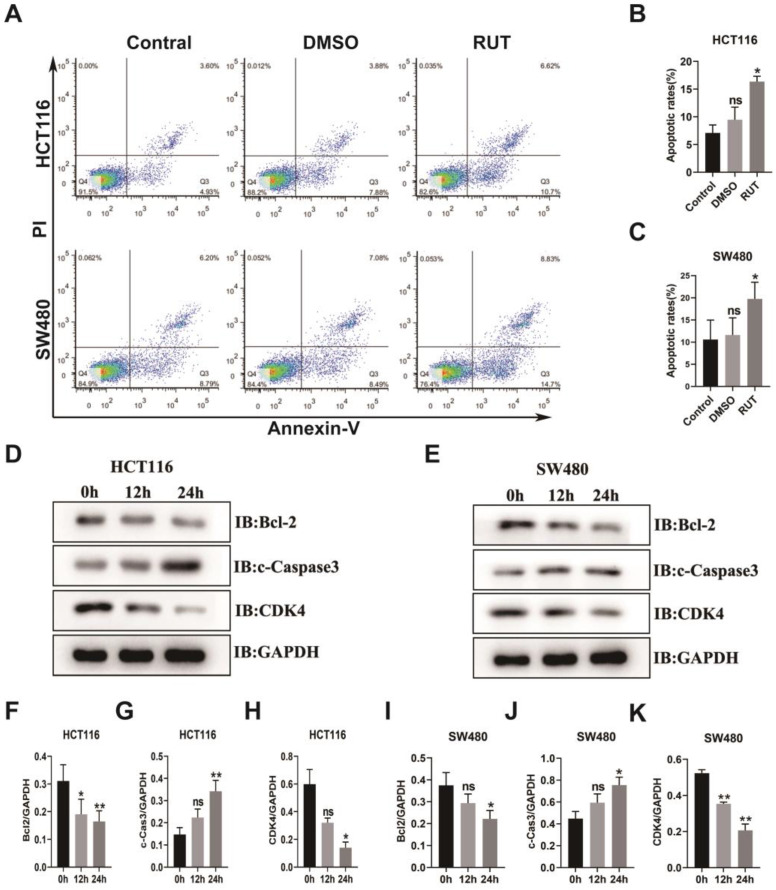
** RUT induced the apoptosis of CRC.** (A-C) CRC cells were treated with RUT (5 μM) or DMSO for 24 h and analyzed using a flow cytometer. RUT significantly induced the apoptosis of CRC (17.32% in the HCT116 RUT treatment group vs 8.53% in the control group; and 23.53% in the SW480 RUT treatment group vs 14.99% in the control group, respectively). (n=3). (D-E) Western blotting results of apoptosis-associated proteins after RUT (5 μM) treatment for 0 h, 12 h, and 24 h. (n=3). (F-K) Statistical results of the protein levels indicated in D and E. ns means no statistically significant difference, *P < 0.05, **P < 0.01.

To elucidate the function of RUT in the apoptosis of CRC cells, we carried out a flow apoptosis experiment. As shown in Figure 4. A-C, the early and late apoptosis rates of the CRC cell lines, HCT116 and SW480, in the treatment group were higher than those in the control group (17.32% in the HCT116 RUT treatment group compared with the control group [8.53%]; and 23.53% in the SW480 RUT treatment group compared with the control group [14.99%]). To determine the functional mechanism of the RUT-induced apoptosis of CRC cells, western blotting was performed to investigate the alteration in apoptosis-related proteins. Our results showed that after RUT treatment, the expression level of the pro-apoptotic protein, cleaved-Caspase3, in both HCT116 and SW480 cells was higher than that after control treatment (Figure 4. D and E). Previous studies demonstrated that the activation of STAT3 induces the expression of antiapoptotic genes, such as Bcl2 and Bcl-xl. Accordingly, CRC cells treated with RUT inhibited the expression of Bcl2 (Figure 4. D and E). However, Rut has no effect on the expression level of the proliferation gene c-Myc (Fig. S2). Therefore, our results show that RUT promotes the apoptosis of colon cancer cell lines by up-regulating the expression of cleaved-Caspase3 and down-regulating the expression of Bcl-2.

### RUT inhibits the growth of CRC cells *in vivo*

**Figure 5:**
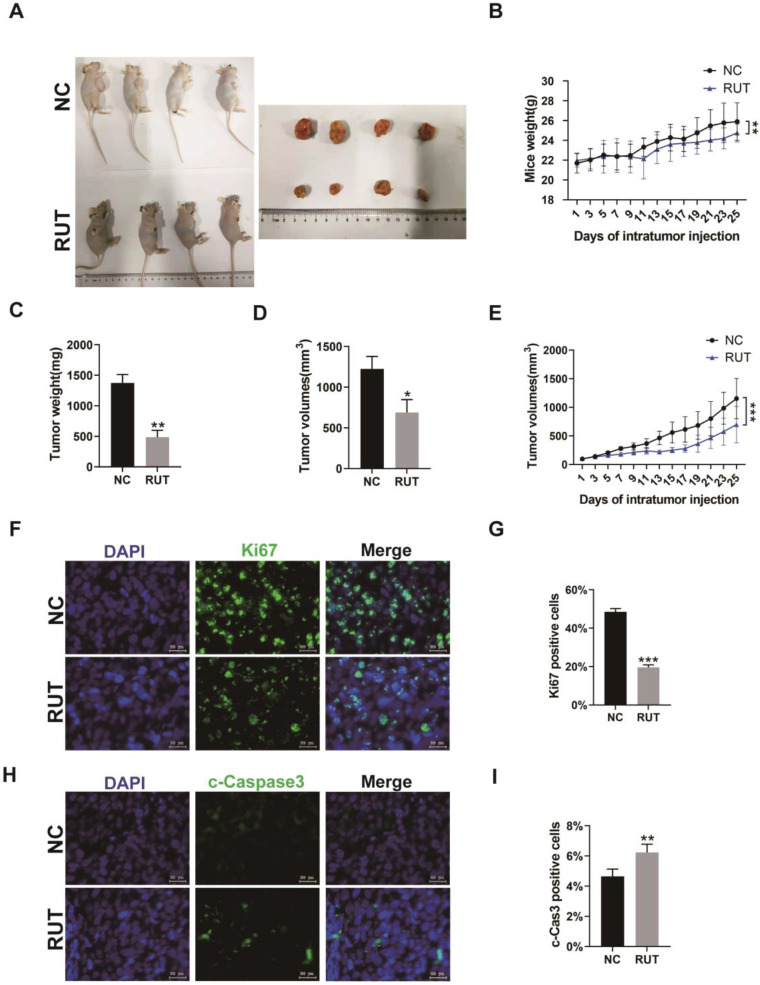
** Effect of RUT on tumor inhibition in vivo.** (A) Images depicting tumor growth in the human CRC cells, HCT116 (2×10^6^ cells), injected into nude mice. (n=6). (B) Mice body weight at different time points. (C) Tumor weight. (D-E) Tumor volume at different time points. (F-I) Immunofluorescence staining for Ki67 or cleaved-Caspase3 in tumor tissues from nude mice with or without RUT treatment (Scale bar: 20 μm). *P < 0.05, **P < 0.01, ***P < 0.001.

To explore whether RUT affects the proliferation of CRC cells *in vivo*, HCT116 cells were subcutaneously injected into BALB/c nude mice to generate xenograft tumors. After tumors reached an average size of 100 mm^3^, mice were injected at 2-day intervals for 4 weeks with vector or RUT. Our result revealed a significant decrease in tumor size (Figure 5. A), tumor weight (Figure 5. C), tumor volume (Figure 5. D and E), and an increase in apoptosis in xenografts from RUT-treated mice. Furthermore, the number of Ki67 positive cells markedly decreased in the tumor tissues treated with RUT compared with those in the control group (Figure 5. F and G), whereas the percentage of cleaved-Caspase3 positive cells in xenografts after RUT treatment was higher than those in the control group (Figure 5. H and I). Altogether, our results suggest that RUT may inhibit the growth of CRC cells* in vivo* by up-regulating the expression of cleaved-Caspase3 and down-regulating the expression of Ki67.

### RUT inhibits CRC lung metastasis

**Figure 6:**
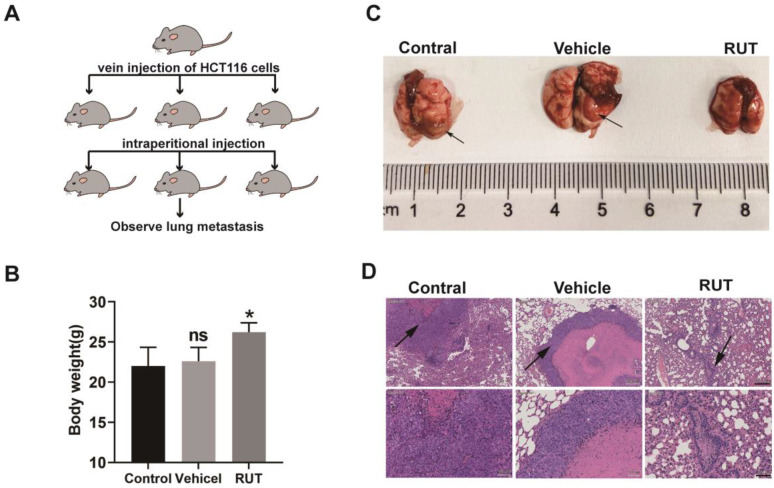
** RUT inhibited CRC lung metastasis.** (A) Images of human CRC cells, HCT116 (5×10^6^ cells), injected into nude mice via the tail vein to establish the lung metastasis models. (n=6). (B) Mice body weight with or without RUT treatment after 8 weeks. (C) Lung metastatic nodules in the untreated and treated nude mice (arrows). (D) Histological section of lung metastatic foci. (Scale bar: 200 μm and 50 μm). ns means no statistically significant difference, *P < 0.05.

To further explore whether RUT inhibits tumor metastasis *in vivo*, we developed a lung metastasis model by injecting HCT116 cells into the lateral tail vein of nude mice (Figure 6. A). The mice treated with vehicle lost more body weight as compared to the RUT treatment group (Figure 6. B). The group that received RUT treatment exhibited fewer metastatic lesions, and the control and vehicle group showed greater tumor metastasis compared with the RUT treatment group (Figure 6. C-D). These results indicate that RUT significantly inhibites CRC cell metastasis to the lung.

## Discussion

CRC is one of the most common gastrointestinal tumors, with a variety of treatment options. However, the incidence of CRC is gradually increasing, the problem of chemotherapy resistance is increasingly prominent, and its mortality rate remains high. Several studies have demonstrated that some natural plants, either alone or in combination, play a critical role in the treatment of cancer 27-29. RUT is an important component of the traditional Chinese medicine, *Evodia rutaecarpa*. Previous studies have reported that RUT exhibits anti-tumor potential through different pathways. For example, RUT improved the immune function to inhibit the growth of prostate cancer cells by increasing the number of CD4^+^ and CD8^+^ cells in peripheral blood 16. RUT also induced the apoptosis of gastric cancer cells by upregulating the expression of Caspase-3 and Bax and downregulating the expression of Bcl-2 19. Other studies have reported that RUT, which is speculated to be a potential cancer treatment drug, inhibited the growth of new blood vessels by mediating the VEGF signaling pathway 30. However, a study on the effect of RUT on CRC cells and its mechanism of action has rarely been reported.

In this study, RUT displayed less cytotoxicity than the clinical chemotherapy drug, 5-fluorouracil, at the same concentration. Herein, we revealed that RUT significantly inhibited the proliferation and growth of the CRC cell lines, HCT116 and SW480, *in vitro*. Furthermore, our data demonstrated that after treatment with RUT, the migration ability and penetration ability of CRC cells were significantly decreased. Previous studies have identified that RUT improved inflammation responses and gross gastric damage by suppressing the NF-κB pathways 13. The role of the NF-κB/STAT3 inflammatory signaling pathway in CRC has been extensively studied. Moreover, it has been confirmed that the activation of NF-κB and STAT3 and their interaction can regulate the proliferation, invasion, apoptosis, and angiogenesis of tumor cells 24-26, 31. NF-κB is well-known to have a prominent role in colorectal tumorigenesis. In fact, our data demonstrated that RUT inhibited the phosphorylation levels of NF-κB p65 in the colon cancer cell lines, thereby aligning with those of previous reports 13. In addition to the NF-κB signaling pathway, the transcription factor, STAT3, is particularly crucial in the development of CRC 32, 33. Activated STAT3 results in the induction of anti-apoptotic expression genes, such as Bcl2 or Bcl-xL, and proliferation-related genes, such as cyclin D1 or c-Myc 34. According to previous evidence, Evodiamine (EVO), the analog of RUT, promotes cell apoptosis and suppresses liver tumor growth by inhibiting the STAT3 signaling pathway 35. Herein, treatment with RUT was also found to inhibit the activation of STAT3 and decrease the expression of Bcl2 in CRC.

The present findings also revealed a significant decrease in tumor size and weight in xenografts from RUT-treated mice. Based on our results, RUT's anti-tumor effects are associated with a decrease in the Ki67-positive cell population and an increase in the number of cleaved -Caspase3-positive cells.

Previous studies have indicated that RUT has an anti-apoptotic function 13, 36. However, we found that Rut remarkably induced the apoptosis of CRC. In this study, we examined several markers for apoptosis to explain the mechanism employed by RUT to induce apoptosis. The results showed that the expression levels of cleaved Caspase-3 increased after RUT treatment. Such finding is similar to the reported mechanism that RUT can activate Caspase-3, upregulate the expression of Caspase-3 and Bax, and downregulate the expression of Bcl-2, thereby promoting the increase in apoptosis of the gastric cancer cell, SGC 7901 19. However, a recent study reported that RUT appears to activate Bcl-2 in the PI3K/Akt-dependent pathway while downregulating Caspase-3 expression and protecting gastric mucosal cells from ethanol damage 36. Such finding suggests that the function of RUT varies in different cell types or in different diseases. However, more studies are needed. Altogether, we demonstrate that RUT exerts its tumor inhibitory effects by inducing apoptosis.

Several studies have reported that RUT is an anti-cancer agent as it inhibits metastasis *in vitro* and *in vivo* 22. In the nude mouse model of colon cancer lung metastasis employed herein, lung nodules were more obvious in nude mice treated intraperitoneally with solvent, while lung nodules were almost invisible in nude mice treated with RUT. These results suggest that RUT may inhibit the metastasis of colon cancer cells *in vivo*.

## Conclusion

**Figure 7:**
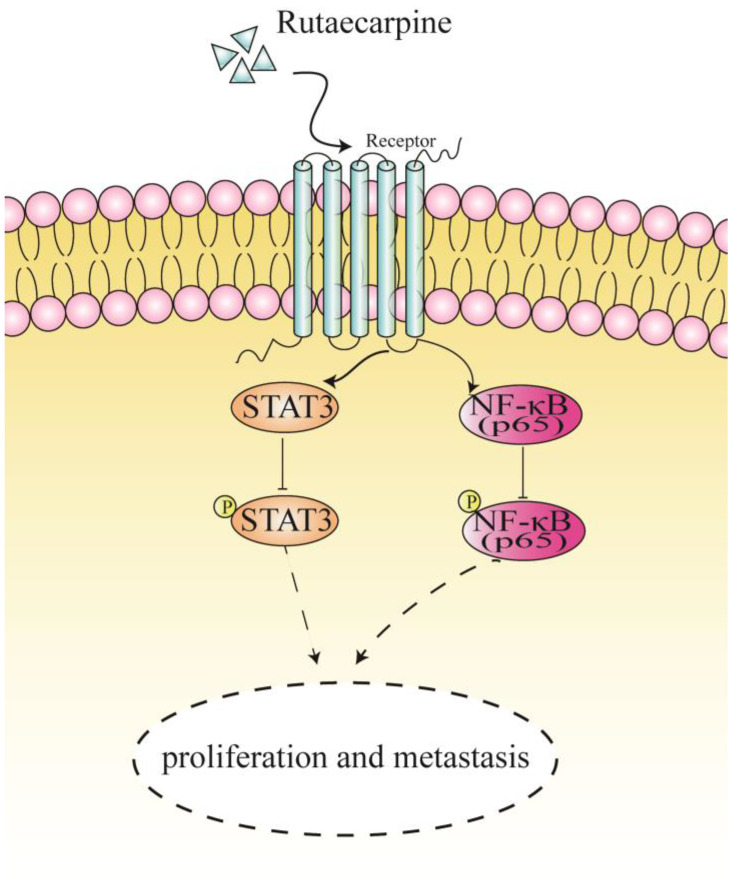
A proposed mechanism of RUT in suppressing the proliferation and metastasis of CRC cells.

In summary, our findings revealed that RUT plays an inhibitory role in the proliferation, migration, and invasion of CRC cells *in vivo* and *in vitro*. Moreover, we demonstrated that RUT inactivates the NF-κB/STAT3 signaling pathway in CRC cells (Figure 7). Our findings highlight a potential approach for the clinical treatment of CRC.

## Supplementary Material

Supplementary figures and tables.

## Abbreviations

CRC: colorectal cancer
RUT: rutaecarpine
NF-κB: nuclear factor kappa-B
STAT3: signal transducer and activator of transcription 3
PI3K: phosphoinositide 3-kinase
NOX4: NADPH oxidase 4
Bcl-2: B-cell lymphoma-2
Bax: BCL2-Associated X protein
VEGF: vascular endothelial growth factor
