# Supplementary material for: Rutaecarpine suppresses the proliferation and metastasis of colon cancer cells by regulating the STAT3 signaling
Source: J Cancer. 2022 Jan 1;13(3):847–57. doi: 10.7150/jca.66177 (PMC8824880; doi:10.7150/jca.66177)
Supplement: Supplementary file 1 — Supplementary figures and tables. [file jcav13p0847s1.pdf]

Supplementary Materials

1.1 Supplementary Figure 1

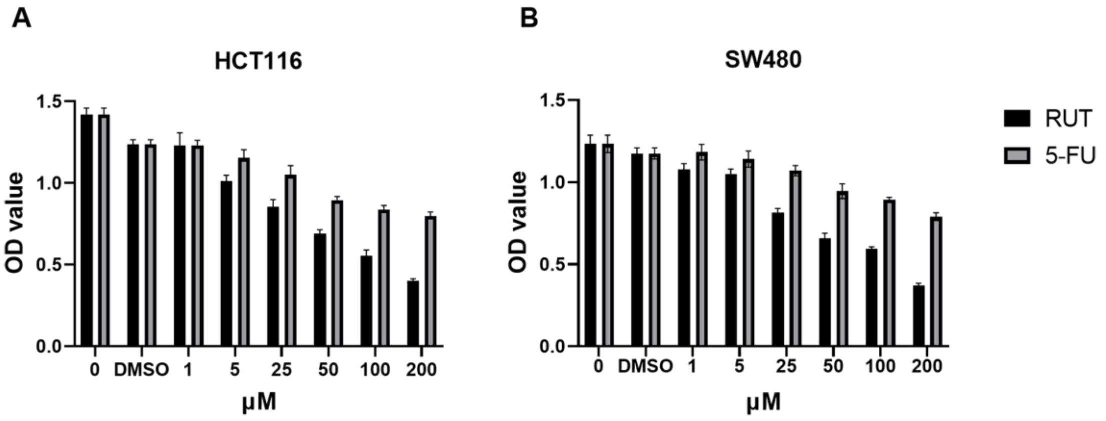

Fig. S1. The effects of RUT and 5-FU on colorectal cancer cell lines (HCT116 and SW480).

1.2 Supplementary Figure 2

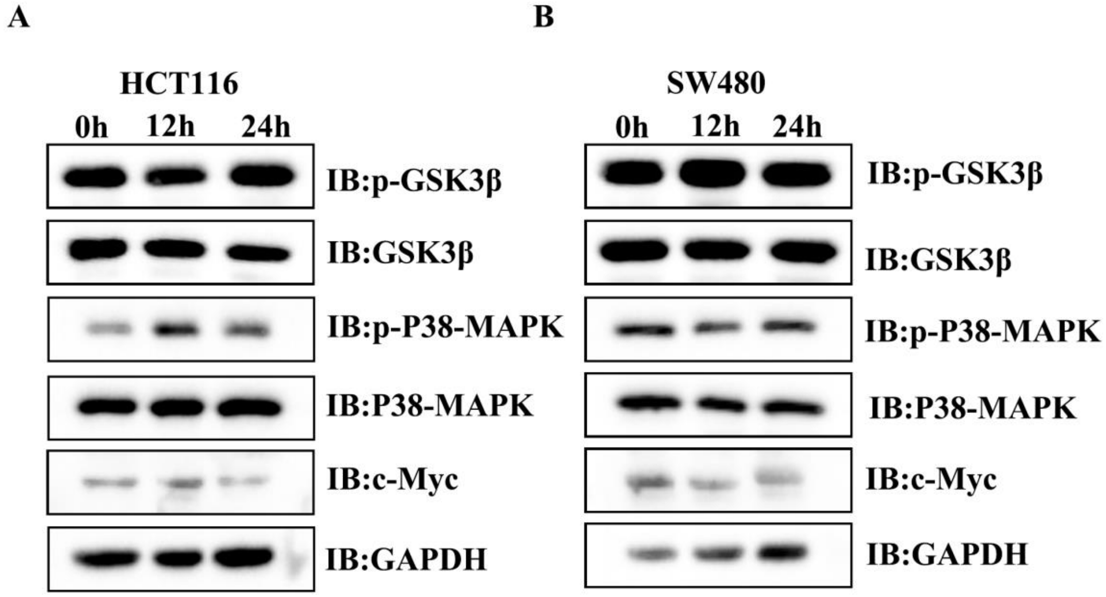

Fig. S2. The difference of protien expression in CRC cells (HC1116 and SW480) with or without RUT trearment.

(A-B) The level of phosphorylated GSK-3β, P38 and the protein levels of c-Myc in HCT116 (A) and SW480 (B) cells after RUT (5 μM) treatment for 0h, 12h and 24 h.
